# Widespread intron retention diversifies most cancer transcriptomes

**DOI:** 10.1186/s13073-015-0168-9

**Published:** 2015-05-15

**Authors:** Heidi Dvinge, Robert K. Bradley

**Affiliations:** Computational Biology Program, Public Health Sciences Division, Fred Hutchinson Cancer Research Center, Seattle, WA USA; Basic Sciences Division, Fred Hutchinson Cancer Research Center, Seattle, WA USA

## Abstract

**Background:**

Somatic mutations affecting components of the RNA splicing machinery occur with high frequencies across many tumor types. These mutations give rise to distinct alterations in normal splice site and exon recognition, such as unusual 3′ splice site preferences, that likely contribute to tumorigenesis.

**Methods:**

We analyzed genome-wide patterns of RNA splicing across 805 matched tumor and normal control samples from 16 distinct cancer types to identify signals of abnormal cancer-associated splicing.

**Results:**

We found that abnormal RNA splicing, typified by widespread intron retention, is common across cancers even in the absence of mutations directly affecting the RNA splicing machinery. Almost all liquid and solid cancer types exhibited frequent retention of both alternative and constitutive introns relative to control normal tissues. The sole exception was breast cancer, where intron retention typified adjacent normal rather than cancer tissue. Different introns were preferentially retained in specific cancer types, although a small subset of introns enriched for genes encoding RNA splicing and export factors exhibited frequent retention across diverse cancers. The extent of intron retention correlated with the presence of *IDH1* and *IDH2* mutations in acute myeloid leukemia and across molecular subtypes in breast cancer. Many introns that were preferentially retained in primary cancers were present at high levels in the cytoplasmic mRNA pools of cancer cell lines.

**Conclusions:**

Our data indicate that abnormal RNA splicing is a common characteristic of cancers even in the absence of mutational insults to the splicing machinery, and suggest that intron-containing mRNAs contribute to the transcriptional diversity of many cancers.

## Background

The discovery of high-frequency mutations affecting components of the RNA splicing machinery is one of the most unexpected results of cancer genome sequencing. ‘Spliceosomal mutations’ are enriched in diverse diseases, including myelodysplastic syndromes, lymphoid leukemias, and solid tumors of the lung, breast, pancreas, and eye, and most commonly cause specific missense changes to the SF3B1, SRSF2, and U2AF1 proteins [1–10]. Mechanistic studies revealed that *U2AF1* mutations alter the preferred 3′ splice site sequence both *in vivo* and in *vitro*, thereby influencing genome-wide recognition of alternative and constitutive 3′ splice sites [11–13]. *SRSF2* mutations similarly alter interactions between SRSF2 and pre-mRNA, resulting in altered exon recognition that promotes dysplastic hematopoiesis [14].

In addition to the direct genetic link between abnormal RNA splicing and tumorigenesis provided by point mutations affecting the spliceosome, indirect evidence suggests that important differences distinguish RNA splicing in normal versus cancerous cells even in the absence of these mutations. Small molecules that inhibit splicing have antitumor activity [15, 16]; the SF3b component PHF5A is differentially required for constitutive splicing in glioblastoma versus normal neural stem cells [17]; RNA splicing is reportedly noisier in cancers than normal cells [18]; increased intron retention is associated with *SETD2* mutations in kidney cancer [19] and castration resistance in prostate cancer [20]. These and other studies together suggest that common RNA processing differences may distinguish cancer and normal cells irrespective of tissue of origin. However, this hypothesis has not been systematically tested.

Here, we took advantage of the comprehensive transcriptome data produced by The Cancer Genome Atlas (TCGA) to identify large-scale differences in RNA splicing between tumor and normal control samples across 16 distinct cancer types. While we observed no obvious biases in cassette exon recognition or 5′ or 3′ splice site recognition, almost all analyzed cancer types exhibited increased levels of intron retention relative to normal controls. The sole exception was breast cancer, for which intron retention characterized normal breast rather than cancer samples. Our results indicate that intron retention is a common correlate of tumorigenesis, and suggest that an abundance of intron-containing mRNAs in cancer cells may increase the diversity of many cancer transcriptomes.

## Methods

### RNA-sequencing data

Unprocessed RNA-seq reads from TCGA were downloaded from CGHub, using all solid tumors with patient-matched samples from the adjacent normal tissue, as well as unmatched acute myeloid leukemia (AML) and breast cancer samples (the unmatched breast cancer samples were only used for the subgroup analysis involving all 1,080 cancer patients). Samples were identified using cgquery v2.1, with ‘state = live’, ‘library_strategy = RNA-Seq’, and ‘sample_type = 0*’ or ‘sample_type = 1*’ for cancer and normal samples, respectively, and the sequence data were downloaded with the GeneTorrent client software. For samples extracted from CGHub prior to November 2013, the raw reads were extracted in BAM format and converted to FASTQ format using sam2fastq v1.2 from UNC Bioinformatics Utilities. For samples extracted after November 2013, the reads were available directly in FASTQ format. All samples were sequenced using the Illumina Genome Analyzer or HiSeq, and reads were from unstranded paired-end libraries, with the exception of a subset of the uterine corpus endometrial carcinoma samples, which were single-end. Samples where the sequencing protocol was ‘TotalRNASeqV2’ were excluded, in order to include only poly(A)-selected RNA-seq libraries.

RNA-seq reads from four healthy bone marrow donors were obtained from the NCBI Gene Expression Omnibus (GEO) under accession number GSE61410 [21]. The library characteristics of these samples match those of the AML RNA-seq samples (average read count: 75 M; paired-end libraries; with read length 2×49 or 50 nt). Subcellular fractionation RNA-seq reads from MCF-7 and K562 cells were obtained from GEO under accession number GSE30567 [22], and restricted to poly(A)-selected libraries. RNA-seq data from breast cancer cell lines were obtained from GSE52643 [23] and GSE48213 [24].

### Genome annotations

Alternative splicing events were classified as cassette exons, competing 5′ and 3′ splice sites, and retained introns, using annotations from MISO v2.0 [25]. Constitutive splice junctions and introns were defined as junctions that were not alternatively spliced in any isoform of the UCSC knownGene track [26]. RNA annotation files were created for transcripts and splice junctions individually, to map the RNA-seq reads to each annotation set separately. The RNA transcript annotation is a combination of isoforms from MISO v2.0 [25], UCSC knownGene [26], and the Ensembl 71 gene annotation [27]. The RNA splice junction annotation was created using an enumerating of all possible combinations of annotated splice sites as previously described [17].

### RNA-seq read mapping

All RNA-seq reads were processed using a standardized pipeline. Step 1: Map all reads to the UCSC hg19 (NCBI GRCh37) human genome assembly using Bowtie v1.0.0 [28] and RSEM v.1.2.4 [29] modified to call Bowtie with the -v 2 mapping strategy and invoked with the arguments --bowtie-m 100 --bowtie-chunkmbs 500 --calc-ci --output-genome-bam on the gene annotation file. Step 2: Filter the resulting BAM file to: (1) remove alignments with mapq scores of 0; and (2) require a minimum splice junction overhang of 6 bp. Step 3: Align all previously unaligned reads to the splice junction annotation file with TopHat v2.0.8b [30] invoked with the arguments --bowtie1 --read-mismatches 3 --read-edit-dist 2 --no-mixed --no-discordant --min-anchor-length 6 --splice-mismatches 0 --min-intron-length 10 --max-intron-length 1,000,000 --min-isoform-fraction 0.0 --no-novel-juncs --no-novel-indels --raw-juncs. The parameters --mate-inner-dist and --mate-std-dev were determined by mapping to constitutive coding exons as determined with MISO’s exon_utils.py script. Step 4: Filter the resulting alignments as in step 2. Step 5: Merge the BAM files from TopHat and RSEM to generate a final set of all read alignments.

### Classification of primary breast cancer samples into the intrinsic subtypes

RNA transcript levels from the 50 genes included in the PAM50 classifier [31] were normalized using the trimmed mean of M values (TMM) method [32] with the scaling factors calculated based on protein-coding transcripts only. The scaled centroids from pam50.robust in the ‘genefu’ R package were used to cluster all tumor samples into the five intrinsic molecular subtypes using intrinsic.cluster.predict with parameter do.prediction.strength = TRUE. The resulting subtype probabilities were assessed to confirm that less than 5 % of samples had a maximum probability below 0.5.

### Identification of differentially spliced isoforms

MISO v2.0 [25] was used to quantify isoform ratios for all annotated alternative splicing events (cassette exons, competing 5′ and 3′ splice sites, and retained introns). For constitutive junctions and retention of constitutive introns the isoform ratios were estimated using junction-spanning reads, as previously described [17]. Individual tumor samples were analyzed by comparing them directly to the patient-matched normal sample (solid tumors) or to the median across normal bone marrow samples (AML). For each sample pair we restricted the analysis to splicing events that had at least 20 reads supporting either or both isoforms, and that were alternatively spliced in our data. Events were defined as differentially spliced within a sample pair if they satisfied the following criteria: (1) at least 20 relevant reads in both samples; (2) a change in isoform ratio of at least 10 %; and (3) a Bayes factor greater than or equal to 1 for differences in isoform ratios, calculated using Wagenmakers’s framework [33]. Sets of tumor or normal samples were analyzed using a two-sided Wilcoxon rank-sum test with the total number of isoform reads from the test versus the reference set of samples (this method was only used for the mutation/subgroup analyses of AML and breast cancer). Events were differentially spliced if they had: (1) a total of at least 20 reads in both sample sets; (2) a change in isoform ratio of at least 10 %; and (3) a *P* value below 0.01.

### Gene Ontology enrichment analysis

For each cancer type, we identified the parent genes of introns that were differentially retained in at least 20 % of samples. These genes were compared against all protein-coding genes using the R package goseq [34] to test for enrichment of gene ontology (GO) Biological Process terms. The ‘Wallenius’ method was used, the results were corrected for gene length bias, and the resulting false discovery rates were corrected using the Benjamini-Hochberg approach. Only terms with at least two ancestors were tested, and only the most detailed terms were returned by the analysis, to eliminate parent terms associated with generic biological processes.

### Effect of *cis*-acting features

All 5′ and 3′ splice site scores were calculated using the maximum entropy modeling method [35]. Intron lengths and GC content were calculated using the hg19 human genome assembly, and the GC content was averaged across each individual intron.

### Effect of *trans*-acting factors

To calculate the proportion of the variation in intron retention that could be explained by mRNA levels, we used a generalized additive model (GAM) from the R-package mgcv [36] v1.8.3. For each cancer type, we selected the most variable introns, defined as having a standard deviation of changes in intron retention for tumor-normal pairs across all patient samples exceeding 0.1 (AML: 852 introns, breast cancer: 621 introns, colon cancer: 740 introns). We extracted all genes associated with the gene ontology terms ‘mRNA catabolic process’ (GO:0006402), ‘mRNA splicing, via spliceosome’ (GO:0000398) and ‘mRNA transport’ (GO:0051028), and used the log_2_ fold-change of TMM normalized gene expression of the matched tumor-normal pairs. GAM models for all individual intron/mRNA combinations were calculated using a Gaussian distribution with ‘identity’ as the link function, and the method ‘GCV.Cp’ was used for estimating the smoothing parameters of the log_2_ fold-changes for the mRNAs.

### Mutation analysis

Somatic mutations from each AML tumor sample were extracted from cBioPortal using the CGDS-R package. For all genes mutated in more than five samples, intron retention was analyzed in the mutated sample set compared to all wild-type tumor samples.

## Results

### Genome-wide identification of differential splicing

To identify systematic differences in splicing between cancer and normal cells, we analyzed the transcriptomes of 16 solid and liquid tumor types that were sequenced as part of TCGA (Table 1). For the solid tumors, we restricted to samples for which patient-matched adjacent normal tissue was available in order to control for potential genetic differences in splicing, as well as reduce artifacts arising from different handling of unmatched samples [21, 37]. For the acute myeloid leukemia (AML) samples, we used four bone marrow samples from healthy donors as normal controls, as patient-matched controls were not available due to the circulating nature of leukemic cells. To help ensure that we studied the mature mRNA pool rather than pre-mRNA or RNA degradation products, we restricted to samples for which both the cancer and adjacent normal samples were sequenced as poly(A)-selected libraries. A total of 805 matched samples were available for solid cancers originating from the bladder, breast, colon, head and neck, kidney, liver, lung, prostate, rectum, stomach, thyroid, and endometrium, as well as for acute myeloid leukemia (Table 1). We quantified global patterns of splicing across these cancer and normal samples, which were sequenced to an average coverage of 74 million paired-end reads (+/- 22 million), using a database of approximately 125,000 annotated alternative splicing events and approximately 160,000 constitutive splice junctions (see Methods).

**Table 1:** Summary of differential splicing across cancer types

	Patients (n)	Coverage (10^6^ reads)	Cassette exons	Competing 5′ splice sites	Competing 3′ splice sites	Retained introns	Constitutive introns	Constitutive junctions	Retained intron ratio
Acute myeloid leukemia	169	75 (8)	1,219	326	377	973	6,239	841	4.74 (1.2)
Bladder	18	56 (14)	894	170	211	296	552	456	1.83 (2.2)
Breast	104	72 (17)	1,052	210	268	266	612	569	-2.01 (2.1)
Colon	37	58 (18)	597	165	200	307	376	291	3.44 (2.6)
Endometrium	21	35 (16)	619	171	180	296	370	312	0.48 (2.4)
Head and neck	40	81 (26)	829	153	202	115	290	418	0.82 (1.7)
Kidney (chromophobe)	25	82 (12)	941	184	228	207	481	484	1.65 (1.4)
Kidney (renal clear cell)	71	80 (16)	729	149	194	143	380	380	1.42 (1.7)
Kidney (renal papillary cell)	30	85 (20)	730	155	220	184	440	408	1.51 (2.1)
Liver	50	70 (15)	413	90	117	106	200	206	0.82 (2.1)
Lung (adenocarcinoma)	51	43 (28)	517	115	141	176	277	233	0.20 (2.6)
Lung (squamous cell)	50	83 (31)	1,068	190	252	236	609	547	0.44 (2.0)
Prostate	43	75 (11)	607	133	185	130	358	337	1.44 (2.4)
Stomach	30	105 (21)	1,048	200	302	206	886	884	0.99 (1.7)
Rectum	8	53 (14)	680	123	172	310	390	314	4.30 (2.3)
Thyroid	58	83 (11)	533	132	177	174	488	332	0.66 (2.4)

Read coverage, number of differentially spliced isoforms, and bias between intron retention in tumor samples compared to patient-matched normal controls Numbers are medians (standard deviation) across all samples

**Fig. 1:**
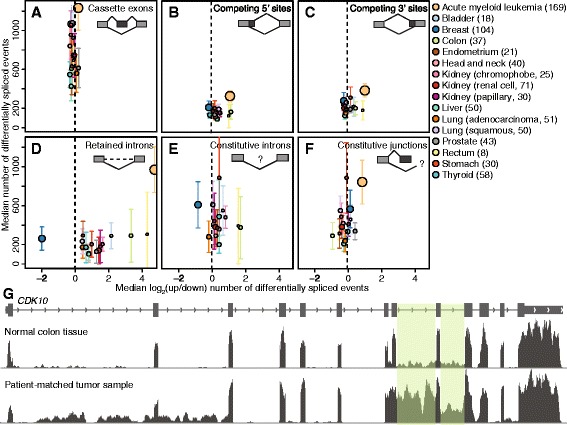
Differential splicing across 16 distinct cancer types. The median numbers of (**a**) cassette exons, (**b, c**) alternative 5′ and 3′ splice sites, and (**d**) retained introns that are differentially spliced between patient-matched cancer and normal samples (y axis) versus log2 of the ratio of the number of events that are up- and downregulated in cancer versus normal controls (x axis). The median is computed across all samples for each cancer type. Upregulation is defined as increased cassette exon inclusion, usage of intron-proximal 5′ or 3′ splice sites, or increased intron retention. Bars indicate the standard deviation across samples for each cancer type, and circle sizes are proportional to the number of samples for each cancer type. See figure for color legend. (**e, f**) As panels A-D, but for (e) retention of constitutive introns or (f) alternative splicing of constitutive junctions. Upregulation is defined as increased constitutive intron retention or decreased alternative splicing of constitutive junctions. For constitutive introns, AML is outside the figure limits (to the right). (**g**) RNA-seq read coverage of *CDK10* for a patient-matched cancer and normal sample from the colon. Shaded boxes mark introns that are most frequently retained in the cancer sample

We first identified annotated alternative splicing events that were differentially spliced in cancer versus normal cells. We defined differentially spliced events as those exhibiting a difference in isoform ratio of ≥10 % between a solid tumor sample and its matched normal, or alternately between an acute myeloid leukemia sample and the median across four normal bone marrow samples. The relative frequency of differential splicing between cancer and normal tissues differed substantially between cancer types, with AML and liver cancers exhibiting the most and fewest differentially spliced events on average. Cassette exons comprised approximately 50 % to 60 % of all differentially spliced events, while competing 5′ and 3′ splice sites and retained introns comprised less than 20 % each across all cancer types (Fig. 1a-d, Table 1), consistent with patterns observed in studies of tissue-specific alternative splicing [38]. Differential splicing of cassette exons and competing 5′ and 3′ splice sites was balanced, in the sense that cassette exons were not preferentially included/excluded and intron-proximal/distal 5′ or 3′ splice sites were not preferentially used.

### Retained introns are common in cancer transcriptomes

In contrast to cassette exon recognition or competing splice site usage, intron retention was markedly imbalanced. All cancer types exhibited a strong enrichment (median of 1- to 20-fold) for increased retention of alternative introns in cancers, with the notable exception of breast cancer, for which adjacent normal tissue exhibited a four-fold enrichment for increased intron retention relative to cancer samples (Fig. 1d). We therefore tested whether constitutive as well as alternative introns were retained. Again requiring a difference in isoform ratio of ≥10 %, we found that constitutively spliced introns were frequently unspliced in cancers relative to normal controls (with the exception of breast cancer), although the enrichment was more modest than for alternative introns (Fig. 1e).

Increased intron retention could potentially be a side effect of the reportedly high levels of noisy splicing in cancers rather than a specific failure to remove intronic sequences [18]. To test this hypothesis, we estimated levels of noisy splicing by quantifying mis-splicing of constitutive junctions, such as splicing from the 5′ splice site of a constitutive junction to a different 3′ splice site of the gene. In contrast to intron retention, where all cancer types except for breast cancer exhibited increased levels of unspliced mRNA, we did not observe uniform increases or decreases in mis-splicing of constitutive junctions in cancer relative to normal transcriptomes (Fig. 1f). Specific introns of a gene were typically retained while others were removed with high efficiency (Fig. 1g).

**Fig. 2:**
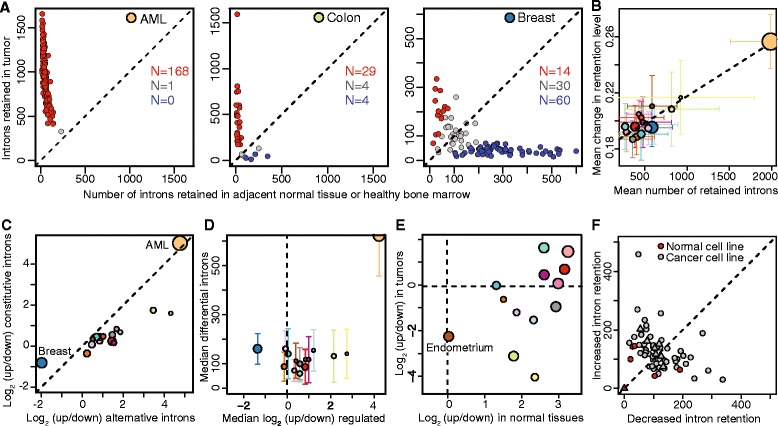
Intron retention characterizes all analyzed cancer types except for breast. (**a**) Numbers of introns with increased or decreased retention in cancer versus control normal samples in AML, colon cancer, and breast cancer. Red/blue illustrate cancer samples that exhibit an enrichment >1.5-fold for increased/decreased intron retention relative to control normal samples. (**b**) The average number of differentially retained introns versus the mean absolute change in retention level, computed for each cancer type. Error bars indicate standard deviation across the individual patient samples. Colors as Fig. 1. (**c**) Median log2 of the ratio of the number of introns that exhibit increased and decreased retention in cancer versus normal controls, where the median is taken over all samples for each cancer type. The x and y axes are, respectively, restricted to annotated alternative and constitutive introns. Colors as Fig. 1. (**d**) As Fig. 1d, but computed using only reads crossing the exon-intron boundary. (**e**) Median log2 of the ratio of the number of introns that exhibit increased and decreased retention in normal breast versus other normal control tissues (x axis), and breast cancer versus other cancer types (y). (**f**) Number of differentially retained introns in breast cancer cell lines (gray) compared to non-cancerous epithelial breast cells (red). Triangles [23]; circles [24]

While intron retention was a readily detectable feature of all cancer types except for breast, the degree of intron retention in any particular sample was highly variable, ranging from increases of 2- to 40-fold in AML and colon cancer (Fig. 2a). The average number of differentially retained introns within a cancer type correlated with the absolute magnitude of the change in intron retention levels (Fig. 2b). The enrichment for retention of alternative versus constitutive introns was well-correlated within each sample for all cancer types (Fig. 2c), suggesting that the propensity to retain introns is a patient-specific characteristic of the transcriptional environment. Here and subsequently, we focused on AML and colon cancer as exemplars of cancers exhibiting strong intron retention, and on breast cancer as the sole example of a cancer exhibiting decreased intron retention relative to normal controls.

As we restricted our analysis to poly(A)-selected cDNA libraries, increased RNA-seq coverage of introns was likely due to the presence of incompletely spliced mRNA rather than inefficiently degraded spliced introns. To confirm that the signal did indeed arise from incompletely spliced mRNA, we quantified intron retention using only reads crossing the exon-intron boundary, which cannot arise from spliced introns or lariats. We observed similar enrichment for intron retention with this conservative measure, indicating that the intronic signal arises from incompletely spliced mRNA (Figs. 1d and 2d).

### Intron retention typifies normal breast tissue instead of breast cancer

Of the 16 cancer types that we studied, only breast cancer was associated with decreased, rather than increased, intron retention relative to normal controls. Infrequent intron retention in breast cancer versus normal breast tissue could be due to efficient intron removal in breast cancer, or instead inefficient intron removal in normal breast. To distinguish between these possibilities, we measured intron retention in breast cancer versus each of the 14 other solid cancer types, as well as in normal breast versus each of the 14 other normal control tissues. We did not observe consistently increased or decreased intron retention in breast cancer versus other cancers. In contrast, there was a strong bias towards increased intron retention in normal breast versus other control tissues, wherein normal breast exhibited more frequent intron retention than any other control tissue except for endometrium (Fig. 2e). Median gene expression of *MKI67*, encoding the proliferation marker Ki-67, was >10-fold higher in breast cancer samples than in adjacent controls, indicating that enhanced intron retention in normal breast is unlikely to be explained by contamination of adjacent control tissue by cancerous cells.

Breast cancer’s status as an outlier in our analysis could potentially be due to the different cell types represented in the cancer versus normal samples. Breast cancer predominantly arises from the mammary ductal or (occasionally) lobular cells, whereas the surrounding stroma consists of fibroblasts, adipocytes, and cells from the immune system. We therefore compared intron retention levels in 50 tumorigenic and six non-tumorigenic breast cell line models [23, 24] (Fig. 2f). While the efficiency of intron removal varied across the cell lines, we did not observe a consistent association between tumorigenic/non-tumorigenic status and degree of intron retention. This cell line analysis is consistent with the hypothesis that cell type differences between tumor and normal samples may contribute to breast cancer’s outlier status, although the known propensity of cell lines to adopt ‘cancer-like’ RNA processing characteristics such as preferential usage of short 3′ UTRs [39] suggests that the same could occur for intron retention.

### Retained introns are frequently specific to the cancer of origin

**Fig. 3:**
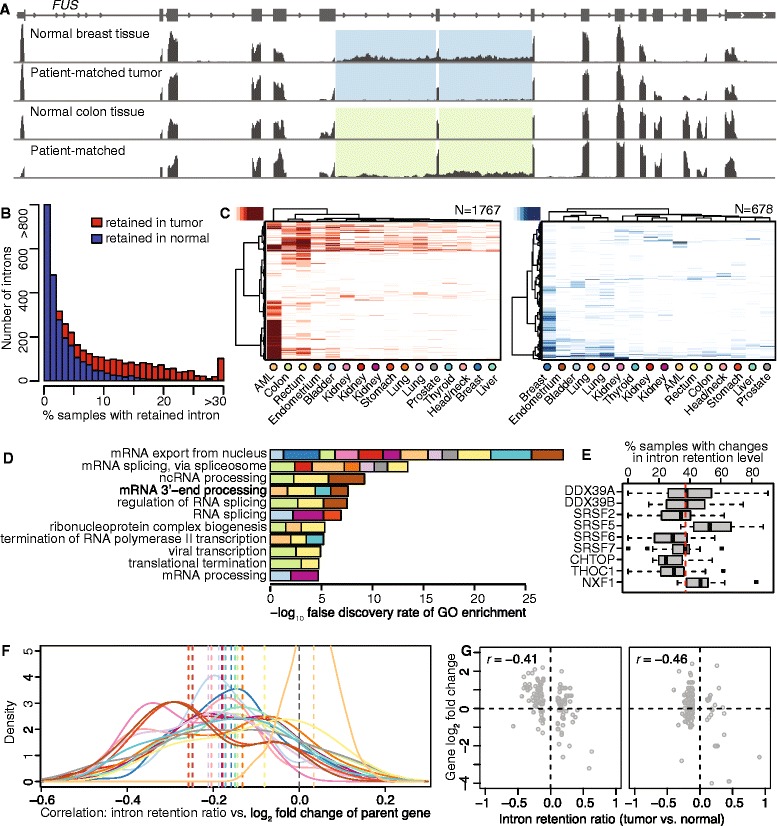
Intron retention preferentially affects genes encoding RNA processing factors. (**a**) RNA-seq read coverage of *FUS* for patient-matched tumor and normal samples from breast and colon. Shaded boxes indicate differentially retained introns. (**b**) Histograms illustrating the numbers of introns exhibiting increased (red) or decreased (blue) retention in cancer relative to normal samples, computed across all cancer types. (**c**) Hierarchical clustering of all retained introns (rows) and all cancer types (columns). Analysis restricted to introns that exhibit increased (red) or decreased (blue) retention relative to normal controls in >10 % of samples for at least one cancer type. Clustering is based on Euclidean distances computed over intron retention frequencies and Ward’s agglomeration method. (**d**) The combined -log_10_ false discovery rate of the most significant Biological Process Gene Ontology (GO) terms enriched among genes containing differentially retained introns in at least 20 % of samples within each cancer type. Colors as in Fig. 1. (**e**) Percentage of samples within cancer types with differential intron retention for select genes mapped to the ‘mRNA export from nucleus’ GO term (GO: 0006406). Dashed line, average across all genes and cancer types. (**f**) Distribution of Pearson correlation coefficients between intron retention and gene expression across all samples within each cancer type. Dashed line, median taken over all samples for each cancer type. Colors as in Fig. 1. (**g**) Scatter plots comparing intron retention to fold-change of the corresponding parent genes for two colon adenocarcinoma samples relative to their patient-matched normal control

Select introns, such as two adjacent introns in *FUS*, were recurrently retained in cancers from many different tissues of origin (Fig. 3a). Quantifying this genome-wide, we found that while most introns were retained in just a few samples of a particular cancer, 1,205 and 171 introns, respectively, exhibited increased or decreased retention in >10 % of cancer samples relative to normal controls across all cancer types (Fig. 3b). An unsupervised cluster analysis confirmed that while some retained introns are shared across most cancer types, the majority are either specific to the cancer of origin, or present at low frequencies in multiple cancers. For example, cancers arising from similar tissues of origin (for example, kidney) clustered together, indicating that they exhibit similar genome-wide patterns of intron retention (Fig. 3c). The preponderance of differentially retained introns in any given cancer sample is typically specific to that cancer, although a subset of introns are frequently retained across diverse cancers.

### Intron retention affects genes involved in RNA processing and nuclear export

We next tested whether genes containing the small subset of introns that are frequently retained across diverse cancers are preferentially involved in particular biological processes (Fig. 3d). We identified Gene Ontology (GO) terms that were enriched for genes exhibiting changes in intron retention affecting at least 20 % of samples within each cancer type. Strikingly, the most enriched terms were all involved in RNA processing, with mRNA export as the most enriched term (Fig. 3d). This association between frequently retained introns and mRNA export is primarily mediated by nine genes that exhibit differential intron retention in more than one-third of all samples on average (Fig. 3e). These genes encode SR proteins, DEAD box proteins associated with the nuclear export factor NXF1, and components of the TREX mRNA export complex.

### Intron retention is modestly anti-correlated with parent gene expression

As intron-containing mRNAs are frequently retained in the nucleus and/or subject to degradation, preferential intron retention in cancer cells could potentially suppress gene expression in the absence of compensatory transcriptional upregulation. Consistent with this hypothesis, a recent study reported that intron retention was associated with lower expression of the parent genes [40], although other studies have reported the opposite trend [20, 41]. We therefore tested whether differential intron retention was associated with altered gene expression by simply computing the Pearson correlation between differences in intron retention and parent gene expression for each cancer sample and matched normal control. For all cancers with the exception of AML, we observed a weak negative correlation between differential intron retention and expression of the corresponding parent genes (Fig. 3f). The origin of AML’s outlier status is unclear, although it may be due to the lack of patient-matched controls. While some tumor-normal pairs exhibited consistent anti-correlation between intron retention and gene expression for many genes, in many cases the anti-correlation was driven by a few outliers with unusually prominent changes in both intron retention and gene expression (Fig. 3g). Our data are consistent with the hypothesis that intron retention contributes to alterations in gene expression [40], although the changes that we observed were generally modest.

### Sequence features distinguishing differentially retained introns

**Fig. 4:**
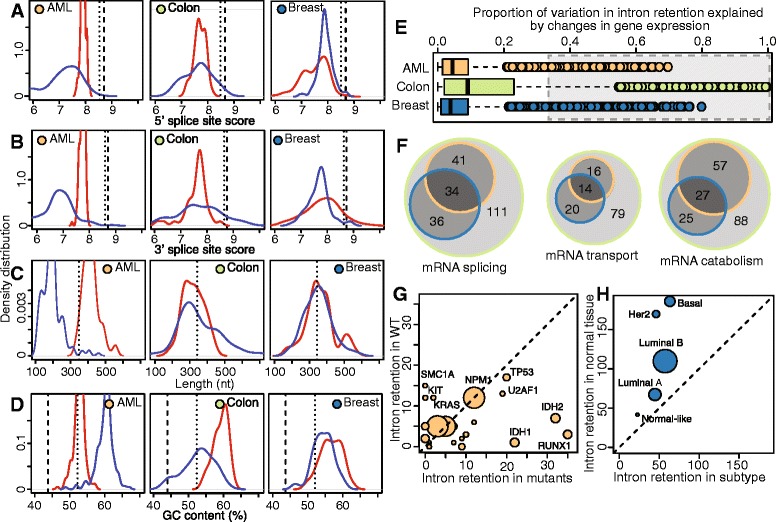
*Cis*-acting sequence features and *trans*-acting factors affect the degree of intron retention. (**a**) 5′ splice site scores, (**b**) 3′ splice site scores, (**c**) length, and (**d**) GC content of introns exhibiting increased (red) or decreased (blue) retention in cancer relative to normal controls. Each distribution illustrates the sample medians per cancer type. Dashed lines, median across all constitutive introns; dotted lines, median across 67,000 constitutive introns <1 kb (resulting in a length distribution similar to alternative introns). The median constitutive intron length of 1.4 kb is outside the range in (c). (**e**) Proportion of variation in intron retention that can be explained by a General Additive Model (GAM) of differential expression of RNA processing, transport and degradation genes. Each dot is a specific intron/mRNA combination. Gray box, combinations where more than one-third of the intron retention level can be explained by expression of *trans*-acting factors. (**f**) Overlap across cancer types for mRNAs within the gray box in (e), divided by Gene Ontology term. (**g**) Numbers of introns exhibiting increased or decreased retention in AML samples mutated in a specific gene compared to all wild-type AML samples for each gene. Dot size is proportional to mutation frequency. Analysis restricted to genes mutated in >5 samples. Mutations with >12 differentially spliced introns are highlighted. Note that the subgroup analyses illustrated here is statistically distinct from previous analyses, which were computed per-sample between matched tumor-normal pairs. This plot only illustrates introns that are consistently differentially spliced across the subgroup relative to other samples. (**h**) Numbers of introns exhibiting increased or decreased retention in breast cancer subtypes relative to all control breast samples (subgroup analysis, as in (g)). Analysis is over all 1,080 cancer and 104 normal breast samples. Dot size is proportional number of samples within each subtype

Previous genome-wide studies have reported that *cis*-acting sequence features distinguish introns susceptible to retention, including weak splice sites, shorter lengths, and higher GC content [40, 41]. Consistent with these studies, introns that were preferentially retained in either normal or cancer samples had weak 5′ and 3′ splice sites, were very short, and had high GC content relative to constitutively spliced introns (Fig. 4a-d). These trends persisted even after controlling for intron length. Trends for introns that were preferentially retained or removed in cancer cells were qualitatively similar, suggesting that similar sequence features predispose introns to inefficient splicing in both normal and cancer cells.

### A subset of retained introns can be explained by expression of RNA processing factors

In addition to *cis*-acting sequence features, we investigated the extent to which *trans*-acting factors may affect differential intron retention. We used a generalized additive model to identify relationships between changes in expression of genes encoding *trans*-acting factors relevant to the RNA life cycle and intron retention. While the majority of RNA processing factors were not systematically predictive of changes in intron retention, expression of a subset of genes was strongly associated with intron retention (Fig. 4e). Many of these genes, which were involved in RNA splicing, transport, and degradation, were associated with intron retention across AML, colon cancer, and breast cancer (Fig. 4f). These genes encoded proteins involved in 3′ splice site selection (for example, *SF3A1*, *SF3B1*, *SF3B2*), the nuclear pore complex (*NUP60*, *NUPL1*, *NPIPA1*, *RANBP2*) and RNA degradation and nonsense-mediated decay (*UPF1*, *SMG1*, *PAN2*, *XRN1*).

### Clinical correlates of intron retention

We next sought to identify clinical variables that potentially contributed to the high levels of inter-sample variability in intron retention that we observed (Fig. 2a, Table 1). We first tested whether specific somatic mutations were associated with enhanced or diminished intron retention relative to normal controls. We focused on AML due to the mounting evidence that presence/absence of specific driver mutations is clinically and therapeutically relevant [42–44]. For each of 22 genes that were somatically mutated in at least five of the 169 AML samples, we computed the number of differentially retained introns in wild-type versus mutant samples. Mutations in most genes were not associated with increased or decreased intron retention relative to other AML samples (although all AML samples exhibited strong intron retention relative to normal bone marrow; Fig. 2a). Mutations in *RUNX1*, *IDH1*, and *IDH2*, in contrast, were associated with a strongly increased intron retention relative to wild-type AML samples (Fig. 4g). For *IDH1* and *IDH2*, the augmented intron retention that we observed is likely an underestimate of the true effect. We treated *IDH1* and *IDH2* separately so that they could serve as a rough validation of each other, and so many of the *IDH1* wild-type samples contained *IDH2* mutations and vice versa.

We next tested whether specific molecular subtypes of tumors exhibited stronger or weaker signals of intron retention. We focused on breast cancer, which is frequently divided into subtypes that correlate strongly with prognosis and response to therapy [45]. Using all 1,080 poly(A)-selected breast cancer samples available in TCGA, we computed the number of differentially retained introns in each molecular subtype relative to all available normal controls (Fig. 4h). (As we performed a subgroup rather than per-sample analysis, we did not restrict to samples with matched tumor-normal pairs.) Normal-like samples exhibited the fewest differentially retained introns relative to all normal controls, consistent with their known similarity to normal breast tissue [45], and also exhibited relatively balanced increases and decreases in intron retention, as did luminal A and B samples. In contrast, basal-like and Her2 amplified or over-expressing samples exhibited the most differential intron retention relative to normal tissue, as well as the strongest bias towards decreased intron retention. As basal-like and Her2 amplified or over-expressing samples are associated with the worst prognosis of the subtypes analyzed, it is interesting to note that a recent study observed high levels of unspliced mRNA in bone metastases from castration-resistant prostate cancer relative to primary prostate cancer [20].

## Discussion

Alternative splicing of specific genes has long been known to contribute to cancer initiation, progression, and metastasis [46–49]. The recent discovery of spliceosomal mutations in diverse cancers, coupled with the identification of spliceosomal components that are differentially required in cancer versus normal cells [17] and the ongoing development of RNA splicing inhibitors as potential antitumor drugs [50–52], suggests that the RNA splicing process may frequently differ substantially between cancer and normal cells. Our study bolsters this conjecture by demonstrating that cancer transcriptomes from diverse tissues of origin frequently exhibit marked increases in intron retention relative to adjacent normal tissue even in the absence of mutational insults to the splicing machinery.

Intriguingly, our simple correlation of clinical variables with intron retention suggested that mutations affecting factors not canonically involved in RNA processing, such as IDH1 and IDH2, may contribute to splicing dysregulation in cancer. While our analysis cannot distinguish between direct and indirect effects, previous reports suggest that aberrant methylation induced by *IDH1* and *IDH2* mutations could indeed alter intron recognition. Exons exhibit preferential DNA methylation relative to introns [53–56] and changes in DNA methylation can influence alternative splicing through differential CTCF binding [57]. We were unable to test whether differentially retained introns in *IDH1* or *IDH2*-mutant samples exhibited differential DNA methylation due to the low probe coverage of introns on the Illumina HumanMethylation450K BeadChip used by TCGA. However, taken together with a recent report that mutations affecting the epigenetic factor SETD2 are associated with loss of histone H3 lysine 36 tri-methylation (H3K36me3) and increased intron retention in clear cell renal cell carcinoma [19], our results suggest that crosstalk between epigenetics and RNA splicing may link a diverse spectrum of mutations to RNA processing defects in cancer.

**Fig. 5:**
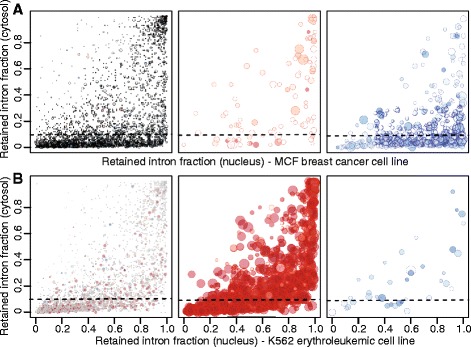
Retained introns are frequently found in the cytoplasm. Intron retention in the nuclear versus cytoplasmic subcellular fractions of MCF-7 (**a**) and K562 (**b**) cell lines, categorized by how frequently the introns are differentially spliced in primary breast cancer or AML samples. Left, introns that are differentially spliced in <25 % of samples from the corresponding primary cancer; center, introns that are retained in more cancer samples than normal controls; right, introns that are retained in more normal controls than cancer samples. The left, center, and right panels are mutually exclusive. Each dot represents a single intron. The dot size is proportional to the number of patients the intron is differentially spliced in, and the dot color intensity represents the proportion of patients where it is retained in the tumor (red) versus the control (blue). Introns lying above the dashed line are retained in >10 % of cytosolic transcripts

While our study and others make increasingly clear that intron retention is much more common in human cells than previously believed, the functional consequences of this intron retention remain unknown. Impaired nuclear export and cytoplasmic RNA degradation both provide barriers to stable expression of intron-containing mRNAs [58–63], and likely reduce many such mRNAs to dead-end products of incomplete splicing. As TCGA data are from whole-cell transcriptomes, we were unable to test whether intron-containing mRNAs are present in the cytoplasmic mRNA pools of primary cancers. We therefore instead used subcellular fractionation data from MCF-7 (breast cancer) and K562 (erythroleukemic) cell lines as rough approximations of the cell types represented in the TCGA primary breast cancer and AML samples [22]. We restricted to introns that were differentially retained in breast cancer or AML relative to normal controls, and quantified the relative abundance of mRNAs containing these introns in the nuclear and cytoplasmic fractions of MCF-7 or K562 cells (Fig. 5). Most intron-containing mRNAs were present at higher levels in the nuclear fraction, as expected, but many retained introns were nonetheless present at high levels in both fractions. Approximately 75 % or 52 % of introns that were differentially retained in breast cancer or AML relative to controls were retained at rates ≥10 % in the cytoplasmic fraction (meaning that the intron-containing mRNA constituted ≥10 % of the mRNAs from the parent gene). With the caveat that data from MCF-7 and K562 cell lines may not closely correspond to *in vivo* processes, we conclude that biased intron retention may generate a diversity of intron-containing mRNAs that are exported to the cytoplasm and sufficiently stable to be readily detectable.

If translated, intron-containing mRNAs could produce novel peptides with unknown consequences, ranging from a potentially adaptive expansion of the proteomic repertoire to a deleterious triggering of tumor immunogenicity. While genes involved in many biological processes are affected by intron retention, it is interesting to note that the small subset of introns that are preferentially retained across many cancers are frequently found in genes encoding RNA processing factors involved in RNA export and splicing (Fig. 3d). Finally, it is tempting to speculate that the apparent abundance of intron-containing mRNAs in tumor cells may contribute to the observed antitumor activity of compounds that inhibit RNA splicing catalysis. Future work will reveal whether intron-containing mRNAs are important contributors to tumor cell biology, host-tumor interaction, and sensitivity to antitumor agents, or instead simply by-products of the widespread molecular dysregulation that accompanies oncogenesis.

## Conclusions

Our data show that intron retention characterizes the transcriptomes of many primary cancers, indicating that globally abnormal RNA splicing is a common correlate of tumorigenesis even in the absence of direct mutational insults to the RNA splicing machinery. Different introns were preferentially retained in specific samples and cancer types, although a small minority of introns were frequently subject to retention in diverse cancers. The degree of intron retention appears to be influenced by a complex interplay of *cis*-acting sequence features and RNA processing factors acting in *trans*, rather than by a single mechanism. Many introns that were preferentially retained in primary cancers were detectable in the cytoplasmic fractions of cancer cell lines, suggesting that at least some intron-containing mRNAs are sufficiently stable to contribute to the transcriptional diversity of cancer cells. Further investigation will determine whether these intron-containing mRNAs are translated into potentially functional proteins, or instead dead-end products of inefficient splicing.

## Abbreviations

AML: Acute myeloid leukemia
GAM: Generalized additive model
GEO: Gene expression omnibus
GO: Gene ontology
TCGA: The Cancer Genome Atlas
TMM: Trimmed Mean of M values

## References

[1] Yoshida K, Sanada M, Shiraishi Y, Nowak D, Nagata Y, Yamamoto R (2011). Frequent pathway mutations of splicing machinery in myelodysplasia. Nature.

[2] Graubert TA, Shen D, Ding L, Okeyo-Owuor T, Lunn CL, Shao J (2012). Recurrent mutations in the U2AF1 splicing factor in myelodysplastic syndromes. Nat Genet.

[3] Papaemmanuil E, Cazzola M, Boultwood J, Malcovati L, Vyas P, Bowen D (2011). Somatic SF3B1 mutation in myelodysplasia with ring sideroblasts. New Eng J Med.

[4] Visconte V, Makishima H, Jankowska A, Szpurka H, Traina F, Jerez A (2012). SF3B1, a splicing factor is frequently mutated in refractory anemia with ring sideroblasts. Leukemia.

[5] Quesada VV, Conde LL, Villamor NN, Ordóñez GRG, Jares PP, Bassaganyas LL (2012). Exome sequencing identifies recurrent mutations of the splicing factor SF3B1 gene in chronic lymphocytic leukemia. Nat Genet.

[6] Imielinski M, Berger AH, Hammerman PS, Hernandez B, Pugh TJ, Hodis E (2012). Mapping the hallmarks of lung adenocarcinoma with massively parallel sequencing. Cell.

[7] Cancer Genome Atlas Network (2012). Comprehensive molecular portraits of human breast tumours. Nature.

[8] Biankin AV, Waddell N, Kassahn KS, Gingras M-C, Muthuswamy LB, Johns AL (2012). Pancreatic cancer genomes reveal aberrations in axon guidance pathway genes. Nature.

[9] Martin M, Maßhöfer L, Temming P, Rahmann S, Metz C, Bornfeld N (2013). Exome sequencing identifies recurrent somatic mutations in EIF1AX and SF3B1 in uveal melanoma with disomy 3. Nat Genet.

[10] Harbour JW, Roberson EDO, Anbunathan H, Onken MD, Worley LA, Bowcock AM (2013). Recurrent mutations at codon 625 of the splicing factor SF3B1 in uveal melanoma. Nat Genet.

[11] Brooks AN, Choi PS, de Waal L, Sharifnia T, Imielinski M, Saksena G (2014). A pan-cancer analysis of transcriptome changes associated with somatic mutations in U2AF1 reveals commonly altered splicing events. PLoS One.

[12] Przychodzen B, Jerez A, Guinta K, Sekeres MA, Padgett R, Maciejewski JP (2013). Patterns of missplicing due to somatic U2AF1 mutations in myeloid neoplasms. Blood.

[13] Ilagan JO, Ramakrishnan A, Hayes B, Murphy ME, Zebari AS, Bradley P (2015). U2AF1 mutations alter splice site recognition in hematological malignancies. Genome Res.

[14] Kim E, Ilagan JO, Liang Y, Daubner MG, Lee S-W, Ramakrishnan A (2015). SRSF2 mutations contribute to myelodysplasia by mutant-specific effects on exon recognition. Cancer Cell.

[15] Nakajima H, Hori Y, Terano H, Okuhara M, Manda T, Matsumoto S (1996). New antitumor substances, FR901463, FR901464 and FR901465, II. Activities against experimental tumors in mice and mechanism of action. J Antibiot.

[16] Kotake Y, Sagane K, Owa T, Mimori-Kiyosue Y, Shimizu H, Uesugi M (2007). Splicing factor SF3b as a target of the antitumor natural product pladienolide. Nat Chem Biol.

[17] Hubert CG, Bradley RK, Ding Y, Toledo CM, Herman J, Skutt-Kakaria K (2013). Genome-wide RNAi screens in human brain tumor isolates reveal a novel viability requirement for PHF5A. Genes Dev.

[18] Chen L, Tovar-Corona JM, Urrutia AO (2011). Increased levels of noisy splicing in cancers, but not for oncogene-derived transcripts. Hum Mol Genet.

[19] Simon JM, Hacker KE, Singh D, Brannon AR, Parker JS, Weiser M (2014). Variation in chromatin accessibility in human kidney cancer links H3K36 methyltransferase loss with widespread RNA processing defects. Genome Res.

[20] Sowalsky AG, Xia Z, Wang L, Zhao H, Chen S, Bubley GJ (2015). Whole transcriptome sequencing reveals extensive unspliced mRNA in metastatic castration-resistant prostate cancer. Mol Cancer Res.

[21] Dvinge H, Ries RE, Ilagan JO, Stirewalt DL, Meshinchi S, Bradley RK (2014). Sample processing obscures cancer-specific alterations in leukemic transcriptomes. Proc Natl Acad Sci U S A.

[22] Djebali S, Davis CA, Merkel A, Dobin A, Lassmann T, Mortazavi A (2012). Landscape of transcription in human cells. Nature.

[23] Sun Z, Asmann YW, Kalari KR, Bot B, Eckel-Passow JE, Baker TR (2011). Integrated analysis of gene expression, CpG island methylation, and gene copy number in breast cancer cells by deep sequencing. PLoS One.

[24] Daemen A, Griffith OL, Heiser LM, Wang NJ, Enache OM, Sanborn Z (2013). Modeling precision treatment of breast cancer. Genome Biol.

[25] Katz Y, Wang ET, Airoldi EM, Burge CB (2010). Analysis and design of RNA sequencing experiments for identifying isoform regulation. Nat Methods.

[26] Meyer LR, Zweig AS, Hinrichs AS, Karolchik D, Kuhn RM, Wong M (2013). The UCSC Genome Browser database: extensions and updates 2013. Nucleic Acids Res.

[27] Flicek P, Ahmed I, Amode MR, Barrell D, Beal K, Brent S (2013). Ensembl 2013. Nucleic Acids Res.

[28] Langmead B, Trapnell C, Pop M, Salzberg SL (2009). Ultrafast and memory-efficient alignment of short DNA sequences to the human genome. Genome Biol.

[29] Li B, Dewey CN (2011). RSEM: accurate transcript quantification from RNA-Seq data with or without a reference genome. BMC Bioinform.

[30] Kim D, Pertea G, Trapnell C, Pimentel H, Kelley R, Salzberg SL (2013). TopHat2: accurate alignment of transcriptomes in the presence of insertions, deletions and gene fusions. Genome Biol.

[31] Parker JS, Mullins M, Cheang MCU, Leung S, Voduc D, Vickery T (2009). Supervised risk predictor of breast cancer based on intrinsic subtypes. J Clin Oncol.

[32] Robinson MD, Oshlack A (2010). A scaling normalization method for differential expression analysis of RNA-seq data. Genome Biol.

[33] Wagenmakers E-J, Lodewyckx T, Kuriyal H, Grasman R (2010). Bayesian hypothesis testing for psychologists: a tutorial on the Savage-Dickey method. Cogn Psychol.

[34] Young MD, Wakefield MJ, Smyth GK, Oshlack A (2010). Gene ontology analysis for RNA-seq: accounting for selection bias. Genome Biol.

[35] Yeo G, Burge CB (2004). Maximum entropy modeling of short sequence motifs with applications to RNA splicing signals. J Comput Biol.

[36] Wood SN (2010). Fast stable restricted maximum likelihood and marginal likelihood estimation of semiparametric generalized linear models. J R Stat Soc.

[37] Dash A, Maine IP, Varambally S, Shen R, Chinnaiyan AM, Rubin MA (2002). Changes in differential gene expression because of warm ischemia time of radical prostatectomy specimens. Am J Pathol.

[38] Wang ET, Sandberg R, Luo S, Khrebtukova I, Zhang L, Mayr C (2008). Alternative isoform regulation in human tissue transcriptomes. Nature.

[39] Sandberg R, Neilson JR, Sarma A, Sharp PA, Burge CB (2008). Proliferating cells express mRNAs with shortened 3′ untranslated regions and fewer microRNA target sites. Science.

[40] Braunschweig U, Barbosa-Morais NL, Pan Q, Nachman EN, Alipanahi B, Gonatopoulos-Pournatzis T (2014). Widespread intron retention in mammals functionally tunes transcriptomes. Genome Res.

[41] Sakabe NJ, de Souza SJ (2007). Sequence features responsible for intron retention in human. BMC Genomics.

[42] Cancer Genome Atlas Research Network (2013). Genomic and epigenomic landscapes of adult de novo acute myeloid leukemia. New Eng J Med.

[43] Smith CC, Wang Q, Chin C-S, Salerno S, Damon LE, Levis MJ (2012). Validation of ITD mutations in FLT3 as a therapeutic target in human acute myeloid leukaemia. Nature.

[44] Dawson MA, Gudgin EJ, Horton SJ, Giotopoulos G, Meduri E, Robson S (2014). Recurrent mutations, including NPM1c, activate a BRD4-dependent core transcriptional program in acute myeloid leukemia. Leukemia.

[45] Sørlie TT, Perou CMC, Tibshirani RR, Aas TT, Geisler SS, Johnsen HH (2001). Gene expression patterns of breast carcinomas distinguish tumor subclasses with clinical implications. Proc Natl Acad Sci U S A.

[46] Shapiro IM, Cheng AW, Flytzanis NC, Balsamo M, Condeelis JS, Oktay MH (2011). An EMT-driven alternative splicing program occurs in human breast cancer and modulates cellular phenotype. PLoS Genet.

[47] Yae TT, Tsuchihashi KK, Ishimoto TT, Motohara TT, Yoshikawa MM, Yoshida GJG (2012). Alternative splicing of CD44 mRNA by ESRP1 enhances lung colonization of metastatic cancer cell. Nat Commun.

[48] Dehm SM, Tindall DJ (2011). Alternatively spliced androgen receptor variants. Endocr Relat Cancer.

[49] Oltean S, Bates DO (2014). Hallmarks of alternative splicing in cancer. Oncogene.

[50] Bonnal S, Vigevani L, Valcárcel J (2012). The spliceosome as a target of novel antitumour drugs. Nat Rev Drug Discov.

[51] O’Brien K, Matlin AJ, Lowell AM, Moore MJ (2008). The biflavonoid isoginkgetin is a general inhibitor of Pre-mRNA splicing. J Biol Chem.

[52] Kaida D, Motoyoshi H, Tashiro E, Nojima T, Hagiwara M, Ishigami K (2007). Spliceostatin A targets SF3b and inhibits both splicing and nuclear retention of pre-mRNA. Nat Chem Biol.

[53] Gelfman S, Cohen N, Yearim A, Ast G (2013). DNA-methylation effect on cotranscriptional splicing is dependent on GC architecture of the exon-intron structure. Genome Res.

[54] Hodges E, Smith AD, Kendall J, Xuan Z, Ravi K, Rooks M (2009). High definition profiling of mammalian DNA methylation by array capture and single molecule bisulfite sequencing. Genome Res.

[55] Chodavarapu RK, Feng S, Bernatavichute YV, Chen P-Y, Stroud H, Yu Y (2010). Relationship between nucleosome positioning and DNA methylation. Nature.

[56] Lyko F, Foret S, Kucharski R, Wolf S, Falckenhayn C, Maleszka R (2010). The honey bee epigenomes: differential methylation of brain DNA in queens and workers. PLoS Biol.

[57] Shukla SS, Kavak EE, Gregory MM, Imashimizu MM, Shutinoski BB, Kashlev MM (2011). CTCF-promoted RNA polymerase II pausing links DNA methylation to splicing. Nature.

[58] Zhou Z, Luo MJ, Straesser K, Katahira J, Hurt E, Reed R (2000). The protein Aly links pre-messenger-RNA splicing to nuclear export in metazoans. Nature.

[59] Huang Y, Steitz JA (2001). Splicing factors SRp20 and 9G8 promote the nucleocytoplasmic export of mRNA. Mol Cell.

[60] Rodrigues JP, Rode M, Gatfield D, Blencowe BJ, Carmo-Fonseca M, Izaurralde E (2001). REF proteins mediate the export of spliced and unspliced mRNAs from the nucleus. Proc Natl Acad Sci U S A.

[61] Le Hir H, Izaurralde E, Maquat LE, Moore MJ (2000). The spliceosome deposits multiple proteins 20–24 nucleotides upstream of mRNA exon-exon junctions. EMBO J.

[62] Kataoka N, Yong J, Kim VN, Velazquez F, Perkinson RA, Wang F (2000). Pre-mRNA splicing imprints mRNA in the nucleus with a novel RNA-binding protein that persists in the cytoplasm. Mol Cell.

[63] Luo MJ, Reed R (1999). Splicing is required for rapid and efficient mRNA export in metazoans. Proc Natl Acad Sci U S A.

